# Influence of the Pneumo-Gastric Nerves on Digestion

**Published:** 1849-07

**Authors:** 


*Influence of the Pneumo-gastric Nerves on Digestion.*—MM. Bouchardat and Saudras give the following results of some experiments performed by them in the *Comptes Rendus*—to determine the influence possessed by the pneumo-gastric nerve over digestion, show clearly that division of both these nerves in the neck at once arrests the process of digestion so far as the stomach is concerned, but has no influence over that part of the process which takes place in the intestines. After feeding dogs with a mixed diet, and then dividing both pneumo-gastric nerves, they found, after twenty-four hours, that those substances, the digestion of which is effected principally in the stomach, such as albumen and fibrine, were quite unchanged, whereas those substances which are digested in the intestines, such as the amylaceous and fatty principles, had been dissolved and absorbed, just as though the pneumo-gastric nerves had been undivided. In several of their experiments, they found that although no chyme is prepared in the stomach after division of the nerves, yet that the starchy principles which pass into the intestine are there converted into glucose, and that the fatty matters are absorbed by the lacteals, just as in the ordinary state of health; so that the digestion and disposal of these principles appear to be quite uninfluenced by the operation. They found, also, that it is not by compression of the trachea by the distended œsophagus, that rabbits die when fed after division of pneumo-gastric nerves as high up as on a level with the larynx.

